# Unexpected rhythm regularity in a patient with atrial fibrillation and a changing frontal plane axis over time

**DOI:** 10.1007/s12471-016-0914-7

**Published:** 2016-10-26

**Authors:** A. Zweerink, E. Leusveld, C. P. Allaart, M. J. B. Kemme

**Affiliations:** Department of Cardiology, VU University Medical Center, Amsterdam, The Netherlands

## Answer

**Fig. 1:**
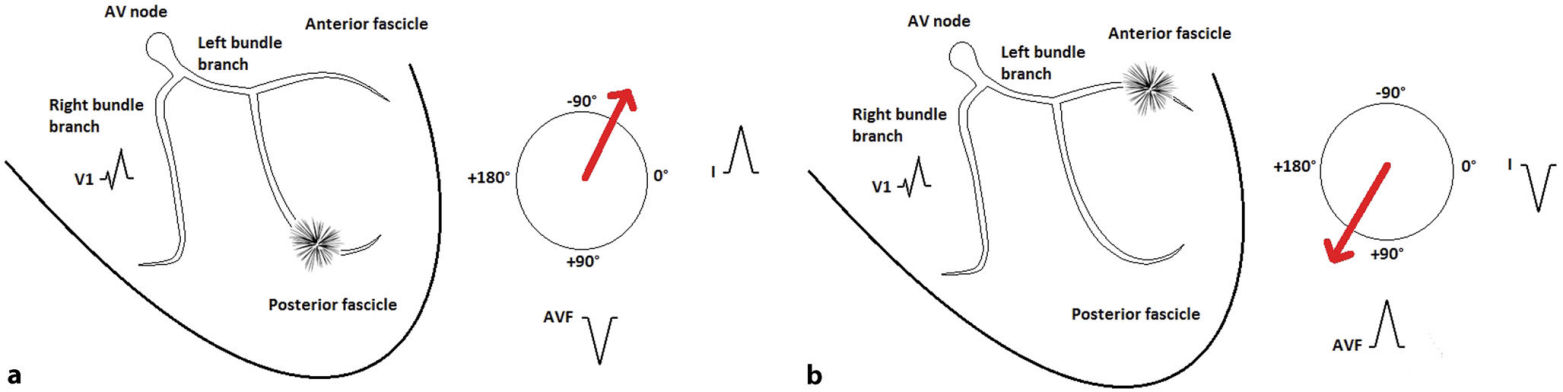
Both figures illustrate a fascicular VT originating from the left bundle branch-Purkinje system, with a focus in the posterior fascicle (**a**) and in the anterior fascicle (**b**), respectively. In both cases the right ventricle is the last to be activated, causing an incomplete RBBB pattern in V1. Impulse formation from the posterior fascicle results in a left-axis deviation (**a**), whereas impulse formation from the anterior fascicle results in a right-axis deviation (**b**)

Both ECGs show a regular, relatively narrow-complex tachycardia of 150 bpm. The first ECG shows an incomplete right bundle branch block (RBBB) pattern in the precordial leads, with the extremity leads demonstrating a left-axis deviation. In the second ECG the frontal plane axis has changed towards a right-axis deviation. Both tachycardias originate from the left bundle branch-Purkinje system causing an incomplete RBBB pattern. In the first ECG, the origin of this fascicular ventricular tachycardia (VT) is located in the posterior fascicle (left-axis deviation) and during the second ECG in the anterior fascicle (right-axis deviation) as illustrated in Fig. 1. In idiopathic fascicular VT, the most likely mechanism is reentry by an excitable gap and a zone of slow conduction [1]. Here, the most likely mechanism is triggered activity in the region of the fascicles due to digoxin intoxication. In this specific case the digoxin level was 3.4 µg/l (reference: max level 2.0 µg/l). As digoxin inhibits the ATPase dependent sodium-potassium pump, increased intracellular calcium concentrations lead to delayed afterdepolarisations and triggered activity [2]. An important feature of the delayed afterdepolarisations is that they can be exacerbated by a shortening of the cycle length and by catecholamines, in this case by atrial fibrillation with rapid ventricular conduction. In rare cases the frontal plane axis alternates on a beat-to-beat basis, this ‘bidirectional VT’ indicates severe digoxin intoxication and is considered life-threatening [3]. Due to its automatic nature, electrical cardioversion is usually not successful. Digoxin-induced fascicular VT is generally responsive to digoxin-specific antibodies with which this patient was successfully treated.
